# Demonstrating Equity Through the Convening of Expert Panels

**DOI:** 10.1089/big.2022.0206

**Published:** 2022-09-06

**Authors:** Tina J. Kauh, Maryam Khojasteh

**Affiliations:** Robert Wood Johnson Foundation, Princeton, New Jersey, USA.

**Keywords:** equity, health, data systems

## Abstract

Population-specific data gaps for a range of demographic characteristics, including race, ethnicity, sex, sexual orientation, gender identity, and disability status, inhibit efforts to protect and improve public health. To identify system and policy levers for addressing these data inequities, the Robert Wood Johnson Foundation (RWJF) convened five expert panels to inform deliberations of the National Commission to Transform Public Health Data Systems (as well as other articles in this supplement). This article reflects the experiences and observations of the authors, RWJF program officers who worked with the expert panels. It provides a brief overview of the process for selecting and convening the expert panels, how this process demonstrated principles of equity, and key themes that emerged across the panels. The processes RWJF used to develop and support the expert panels reflect the Foundation's effort to challenge orthodoxies in research and philanthropy that perpetuate and exacerbate disparities in health and well-being.

## Introduction

Significant data gaps exist for populations based on a range of demographic characteristics, including race, ethnicity, sex, sexual orientation, gender identity, and disability status.^1–3^ The COVID-19 pandemic has shined a bright light on this critical inequity, pointing to the failure of existing data systems to identify disparate risks and impacts of the virus across populations. This failure also affected the ability of state and local communities to provide necessary resources in response to the pandemic, such as guidance for prevention and treatment, testing, medical care, and vaccinations. To help modernize the nation's health data systems, the Robert Wood Johnson Foundation (RWJF) established a first-of-its-kind National Commission to Transform Public Health Data Systems^4^ to reimagine how data are collected, shared, and used, and to identify the investments needed to improve health equity.

To inform the Commission's deliberations, the Foundation supported the development of five expert panels, each focused on identifying barriers and opportunities for improving data systems to help eliminate data gaps for five populations: American Indians and Alaska Natives, Blacks/African Americans, LGBTQ+ communities, people with disabilities, and women. These expert panels served as an opportunity for RWJF to not only inform the Commission but to also put into action the principles it supports around equitable practices. This article provides a brief overview of the process in which the expert panels were selected and convened and how this process demonstrated principles of equity, and a snapshot of key themes that emerged across the five panels. (Importantly, each population has unique issues and concerns that must be considered in the context of creating transformative change in health data systems, and as such, each panel produced reports^5^ with in-depth findings and recommendations.)

## Principles Guiding Equitable Research and Philanthropy

In recent years, philanthropy and the research field have increasingly advocated for greater equity in evaluation, which has historically undervalued the voices, expertise, and capacity of communities on which health interventions focus. This call for change is particularly important, given the potential for practices to perpetuate or exacerbate existing inequities.^6^ The Equitable Evaluation Initiative (EEI) calls for not only evaluators but also philanthropy and other funders to rethink existing evaluation orthodoxies, “deeply held beliefs about ‘how things are done’ that often go unstated and unquestioned.”^7^

While EEI's framework targets the evaluation field, many of these traditional beliefs guide philanthropy and research practices more broadly, including the ideas that (1) researchers should be selected based on credentials that reflect traditional notions of expertise, (2) researchers are the experts and final arbiters, (3) the funder is the primary user of the end product, and (4) findings should provide generalizable lessons. Through the convening of expert panels for the Commission, RWJF aimed to counter these orthodoxies.

## Expert Panel Process

RWJF began developing the expert panels for the Commission by identifying a lead for each panel. That individual was selected based on (1) a clear focus in their work on the panel's target population, (2) demonstrated application of data in their work (either through research, practice, or advocacy), and (3) self-identification as a member of the panel's focus population. Leads had the freedom to select 8–10 members based on their knowledge of the field and their community. The only requisites provided by the Foundation were the same as those used to select the panel leads.

Many leads turned to their peer networks, recognizing colleagues in the research field, as well as those working directly within their communities to advocate for policy change using data or to provide services based on data. Factors like individuals' work in their communities and the extent to which their work prioritized their population's needs were of primary importance in selecting panel members.

The work of the panel was conducted in two phases: Fall 2020/Spring 2021 and Fall 2021/Spring 2022. During Phase 1, each panel convened virtually for a total of 8 hours over 3 days. During Phase 2, each panel had the flexibility to decide when and how often they reconvened. All panel leads and members received honoraria for their participation. Panel convenings were self-facilitated, but RWJF provided logistical support through consultants, who, when possible, shared the panel's identity. These consultants were key partners in building the infrastructure necessary for the panel leaders to stay engaged, informed, and supported with an array of strategic and technical assistance. The ways in which the expert panels were developed and supported throughout the process demonstrate how the Foundation aimed to counter four research orthodoxies.

### Researchers should be selected based on credentials that reflect traditional notions of expertise

Expert panels typically involve leaders in a field who convene to review and synthesize evidence and develop recommendations for next steps based on their expertise. Expertise is defined as having a special skill or knowledge representing proficiency in a particular subject.^8^ Often, convener organizations gauge expertise based on criteria like the number of an individual's academic publications, the prestige of their organizational affiliations, or attainment of an advanced degree.

For the Commission panels, the Foundation recognized that expertise stems from more than an individual's professional credentials. RWJF expanded its definition of expertise by equally valuing an individual's ability to bring a personal lived experience to an issue, one based on being a self-identified member of a particular population (the focus of the panel), in addition to having direct experience in working on data issues across various sectors, including, but not limited to government agencies, organizing and advocacy groups, service providers, and academia.

### Researchers are the experts and final arbiters

The charge to the expert panels was intentionally broad: identify policy and systems-level levers for addressing data gaps regarding the focus population. Panels had the freedom to choose how discussions were facilitated and feedback was elicited, what issues were raised, and the ways in which recommendations were framed. Often, expert panels begin with a prescribed set of questions developed by the funder, and panel members are asked to tell funders the answers. In this study, RWJF began the process by telling the panels members, “You are the experts. You are members of the [focus population]. You tell us what's important to your community and why.”

Each panel then summarized its input into reports shared directly with the Commission to inform its deliberations. The panel on LGBTQ+ youth and adolescents went a step farther by sharing its report with a crucial group of experts, youth in the community, whose feedback helped shape the panel's final product.

### The funder is the primary user of the end product

While the first phase of the expert panels' work involved developing recommendations for the National Commission, the second phase was left open ended, allowing the panel members to choose and pursue follow-on activities that they deemed valuable for them and their community. End products were diverse, and included journal articles, conference presentations, op-eds, and toolkits. They were disseminated in various ways to their respective communities and to produce the most effective policy and systems change, including through social media videos and pieces for regional and national media outlets. The Foundation also provided communication support to facilitate dissemination of the products that were independently pursued by the panels, to help lift up community voice.

### Findings should provide generalizable lessons

While cross-cutting themes are summarized below, the individual in-depth reports for each panel were what were shared with the National Commission to inform its deliberations.

## Summary of Cross-Cutting Themes

Themes that emerged across panels (and thus populations) were synthesized to highlight that the inadequacies of existing data systems (the ways in which data are collected, analyzed, and disseminated) are broad and affect multiple populations. Importantly, many of the themes below are undergirded by the notion of *power*—the belief that whoever holds the data and controls the systems around those data also controls the narrative and has more decision-making power over distribution and use of resources. Currently, the power in data system—and the institutions that build, support, and sustain them—is rooted in dominant white, heteronormative, ableist, and patriarchal values that foster existing systems of oppression and continue to marginalize all those who do not fit into these norms.

As a result, the “dominant” social groups tend to view and understand members of oppressed minority or subpopulation communities through a deficit lens that frames their behaviors and traits as problematic. The expert panels, regardless of whether they focused on race/ethnicity, sexual orientation, gender identity, sex, or disability status, observed that there needs to be a major shifting and redistribution of power to truly effect transformative change in data systems—from conception, to collection, to analysis, to dissemination. The themes below reflect recommendations for how to achieve this.

- Narrative change is critically needed to (1) reframe the purpose of data systems from business, monitoring, and accountability to health, wellness, and equity and (2) reframe the health and well-being of populations to focus on their strengths, assets, and value to society, as opposed to perceived deficits.
- Data and data systems must be decolonized and redefined in ways that are not rooted in dominant white, heteronormative, ableist, and patriarchal culture, recognizing and valuing diverse cultural histories, values, norms, and perspectives.
- Authentic community engagement and partnership with historically marginalized communities, where community members participate in decision-making about data and play a role in providing meaningful oversight of data (e.g., collection, analysis, reporting, and dissemination), are necessary to ensure that data, data systems, methodology, and research questions are relevant and valid.
- Data ownership, where community members have access to and can lead and/or participate in data systems, fosters trust and engagement and transfers power to communities that are the focus of the data.
- Training and capacity building for people, both within and outside a given community, must be a priority so that data and data systems reflect community-specific needs, culture, and history, and so that systems build the pipeline of experts within and leverage existing assets of a population.
- Definitions of terms identifying populations need to be clarified to create greater consistency across data systems.
- Data need to be disaggregated within broad categories of populations to reflect populations' diversity and to acknowledge that they are not a monolith.
- Data systems need to more consistently collect a broader array of demographic variables to allow for analysis of intersectional identities and to reflect the wide range of experiences that impact the health and well-being of the nation's population.
- Technical advances that allow for systems-level interconnectivity, interoperability, and data integration are necessary so that data across systems can communicate with each other and data consumers can access data longitudinally across an individual's life span and link data across a wide range of social determinants of health.

## Conclusions

This article describes RWJF's efforts to employ equitable practices in the convening of expert panels. Each panel consisted exclusively of individuals who brought expertise to the issues based *both* on their work and training with data and, importantly, based on their own personal lived experience as members of the focus community. In this way, the voices and the expertise of focus communities informed the panels' work as they developed recommendations for advancing data systems and data equity. RWJF sought to promote equity by countering research orthodoxies that perpetuate and exacerbate existing inequities in data, and that threaten the well-being of communities, particularly those that already face structural barriers to health and well-being.

## Abbreviations Used

EEI: Equitable Evaluation Initiative
RWJF: Robert Wood Johnson Foundation
